# Directly Infected Resting CD4+T Cells Can Produce HIV Gag without Spreading Infection in a Model of HIV Latency

**DOI:** 10.1371/journal.ppat.1002818

**Published:** 2012-07-26

**Authors:** Matthew J. Pace, Erin H. Graf, Luis M. Agosto, Angela M. Mexas, Frances Male, Troy Brady, Frederic D. Bushman, Una O'Doherty

**Affiliations:** 1 Department of Pathology and Laboratory Medicine, University of Pennsylvania School of Medicine, Philadelphia, Pennsylvania, United States of America; 2 Department of Microbiology, University of Pennsylvania School of Medicine, Philadelphia, Pennsylvania, United States of America; Emory University, United States of America

## Abstract

Despite the effectiveness of highly active antiretroviral therapy (HAART) in treating individuals infected with HIV, HAART is not a cure. A latent reservoir, composed mainly of resting CD4+T cells, drives viral rebound once therapy is stopped. Understanding the formation and maintenance of latently infected cells could provide clues to eradicating this reservoir. However, there have been discrepancies regarding the susceptibility of resting cells to HIV infection *in vitro* and *in vivo*. As we have previously shown that resting CD4+T cells are susceptible to HIV integration, we asked whether these cells were capable of producing viral proteins and if so, why resting cells were incapable of supporting productive infection. To answer this question, we spinoculated resting CD4+T cells with or without prior stimulation, and measured integration, transcription, and translation of viral proteins. We found that resting cells were capable of producing HIV Gag without supporting spreading infection. This block corresponded with low HIV envelope levels both at the level of protein and RNA and was not an artifact of spinoculation. The defect was reversed upon stimulation with IL-7 or CD3/28 beads. Thus, a population of latent cells can produce viral proteins without resulting in spreading infection. These results have implications for therapies targeting the latent reservoir and suggest that some latent cells could be cleared by a robust immune response.

## Author Summary

While HIV is a treatable disease due to effective antiviral therapies, these drugs do not cure HIV. When therapy is stopped, a pool of infected, long-lived, treatment resistant cells re-establishes infection. These latently infected cells, mainly resting CD4+T cells, are barriers to a cure. Studying and understanding the properties of these cells is therefore important to eradicating HIV. It is believed that these latent cells do not produce viral proteins and thus are invisible to the immune system. Here, we show using an *in vitro* HIV model that a population of latently infected cells can produce HIV Gag. Interestingly, this protein production does not result in the release of detectable infectious virus and so the latent cells are unaffected by antiviral therapy. We therefore examined why some latent cells can produce viral proteins without viral spread. We found that resting cells have the ability to make some of the components required for spreading infection but not all are in sufficient quantity. These results have important implications for treating the latent reservoir, as our work suggests that latent cells might be recognized by a boosted immune response.

## Introduction

Highly active antiretroviral therapy (HAART) has been successful in suppressing HIV-1 replication and maintaining CD4+T cell counts in patients. However, long-lived, treatment resistant reservoirs are still a barrier to curing HIV. These latently infected cells are predominantly resting CD4+T cells capable ofreleasing infectious virions after stimulation [1], [2]. A major hurdle in studying HIV latency *in vivo* is the very low frequency ofthese cells. Thus, developing *in vitro* models with a high frequency of latently infected cells is critical to study the establishment, maintenance, and properties of the latent reservoir. Such models in turn can be used to develop therapies to eliminate these cells.

Several *in vitro* latent models using primary cells have been described [3]–[13]. Most of these models rely on activation steps for not only expanding CD4+T cells but also for infection, as several reports have shown blocks to HIV infection in resting CD4+T cells [10], [14]–[19]. While these models can generate sufficient numbers of cells for drug screening [8], [9], they have distinct disadvantages. Activation steps are typically vigorous and result in several changes in cell phenotype [20], [21], some of which narrow the types of CD4+T cell subsets that can be studied *in vitro* [4], [8], [9]. Other models, with less vigorous stimulation steps, avoid these issues but result in low levels of infection [10], [22]. We previously demonstrated that HIV directly integrates into resting CD4+ T cells without requiring any stimulation using a technique called spinoculation [6], [23], [24]. Here, we take advantage of the high level of infection that we can obtain with this method to ask if viral proteins can be expressed in latently infected cells.

Studies *in vivo* have indicated a population of resting cells can transcribe and translate HIV and SIV proteins [25], [26]. These cells were phenotypically resting but were believed to be productively infected and not truly quiescent due to prior activation or their cytokine milieu [25]. Here, we investigate whether latently infected resting CD4+T cells can transcribe and translate viral proteins without stimulation while in a latent state.

## Results

### Spinoculation, IL-7 and CCL19 do not alter the susceptibility of resting CD4+ T cells to HIV integration (Figure 1)

**Figure 1:**
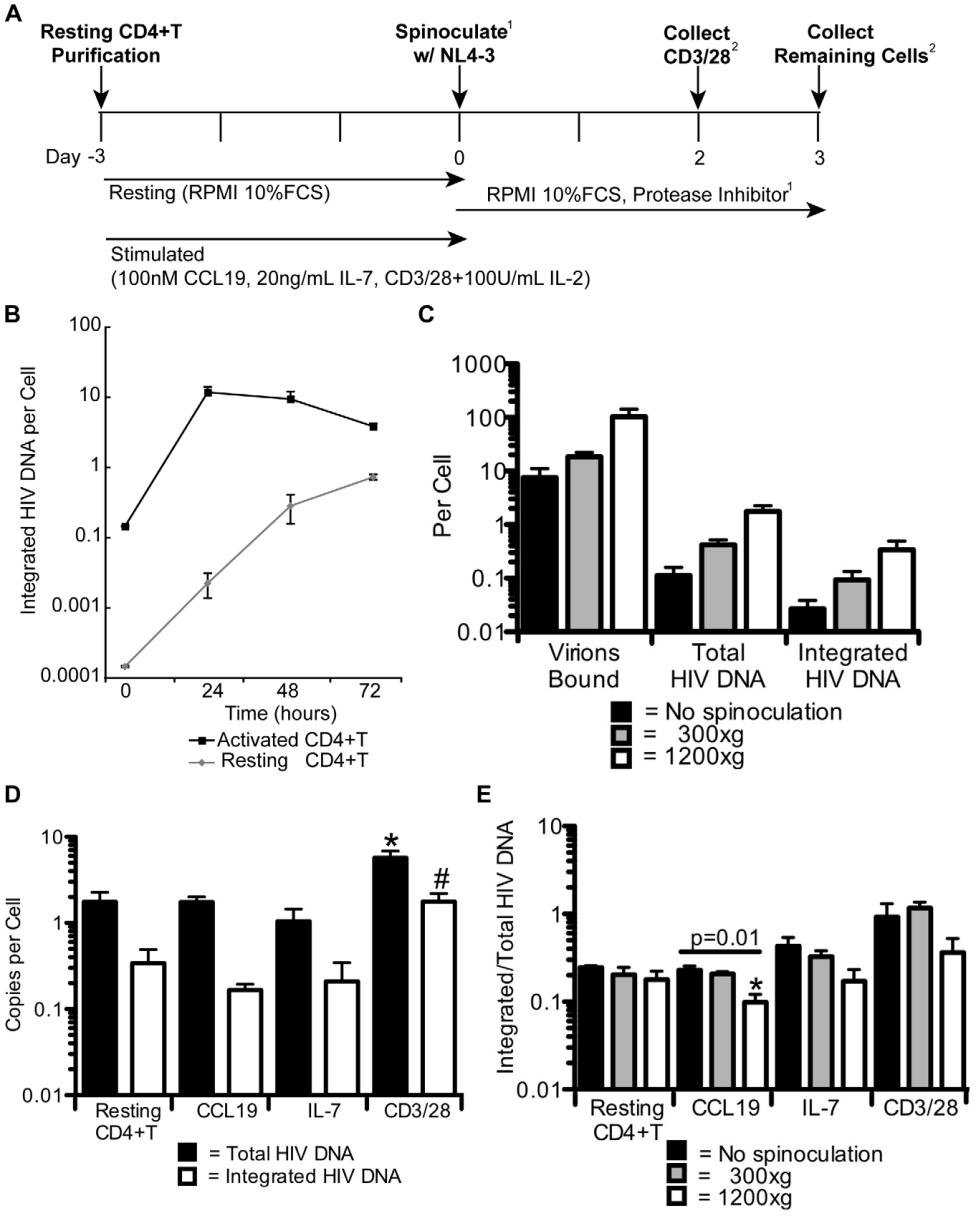
Spinoculation, IL-7 and CCL19 do not alter the susceptibility of resting CD4+T cells to HIV integration. An experimental schematic summarizing all experiments is shown in A. In B, a representative experiment shows the levels of integration measured at 0, 24, 48, and 72 hours post infection in resting and CD3/28 activated CD4+T cells. In C, purified resting cells were infected with HIV (MOI of 3) without spinoculation or were spinoculated at 300×g or 1200×g. Viral binding, total and integrated HIV DNA were measured. The average of 3 experiments in 3 different donors is shown. In D, purified resting cells or cells stimulated with CCL19, IL-7, or CD3/28 beads were spinoculated for 2 hours at 1200×g at an MOI of 3. The average total and integrated levels of 3 donors are shown. In E, cells were treated as in B. Cells were collected as described in A. The average ratio of integrated to total HIV DNA for 3 experiments with 3 different donors is shown. Error bars represent the standard error of the measurements. ^1^ This condition was modified in some later experiments and if so was noted. ^2^ The timepoint changed in some later experiments and if so was noted. *Statistically significant p<0.017. ^#^p = 0.018, not statistically significant due to Holmes correction.

Before we could examine protein expression in latently infected cells, we needed to address concerns regarding the effects of spinoculation on the susceptibility of resting cells to HIV infection. A recent report suggests that spinoculation might induce signaling cascades that artificially allow integration to occur in otherwise resistant resting cells [27]. This evidence is consistent with reports that cytokine stimulation is required for integration to occur in quiescent cells [11], [15]. Therefore, we wanted to determine if spinoculation and/or cytokine treatment enhance integration efficiency in resting CD4+T cells.

To first confirm that integration could occur in resting cells, we spinoculated purified resting CD4+T cells (HLA-DR^−^, CD25^−^, CD69^−^, greater than 98% pure) with HIV (Figure 1A). We have previously shown that this cell population is in the G_0_/G_1a_ stage of the cell cycle [6], [28]. We found that integration occurred in these cells albeit with slower kinetics than activated cells (Figure 1B), consistent with our prior results [29]. Notably, the level of integration in resting cells approached the levels detected in activated cells after 3 days in culture (Figure 1B).

We next wanted to test if spinoculation enhanced infection primarily at the step of viral binding in resting cells rather than at integration as has recently been suggested [27]. We therefore measured viral binding, total HIV DNA, and integrated HIV DNA when resting cells were infected without spinoculation or when they were spinoculated at 300×g or 1200×g. Binding was 3–4 higher in cells spinoculated at 300×g and 13–16 fold higher in cells spinoculated at 1200×g. This increased binding resulted in a similar increase in reverse transcription and integration (Figure 1C). These data indicate that spinoculation enhances HIV infection at the step of viral binding and does not enhance reverse transcription or integration efficiency. Thus, there was no further enhancement downstream of viral binding.

We next tested whether cytokine stimulation would enhance the efficiency of integration in our system as has recently been described in other models [11], [22]. To do this, we cultured untreated resting CD4+T cells or prestimulated the cells for 3 days with IL-7, CCL19, or CD3/28 beads (Figure 1A) and then spinoculated the cells with HIV, after which we measured the ratio of integrated to total HIV DNA 48 hours post infection. We found that the efficiency of integration was similar in all conditions as calculated by the ratio of reverse transcripts that integrate (Figure 1D, E). The level of integration was approximately 5-fold higher in the CD3/28 treated cells (p = 0.018, Figure 1D), but this increase in integration was largely due to an increase in reverse transcription, leaving the integration efficiency unchanged. Thus, treatment with cytokines does not enhance integration efficiency in our model.

We then investigated whether stimulation enhanced integration efficiency when cells were not spinoculated. To test this we infected resting CD4+T cells or cells pre-stimulated with CCL19, IL-7 or CD3/28 beads with HIV (Figure 1A). We compared cells infected without spinoculation to those spinoculated at 300×g and 1200×g by measuring total and integrated HIV DNA. The efficiency of integration was never higher in the spinoculated samples and was similar in most cases (Figure 1D), although CCL19 treatment resulted in slightly lower integration frequency at 1200×g (∼2 fold, p = 0.01, Figure 1D). However, due to the small effect, we refrain from making any conclusions based on this decrease. We note that CD3/28 treated cells trended towards a higher efficiency of integration (Figure 1D), suggesting that integration efficiency may be higher in artificially activated cells. These results indicate that cytokine stimulation did not enhance integration efficiency even without spinoculation.

A final concern regarding our model was the possibility that spinoculation altered the activation state of the resting cells. We therefore assessed the effect of spinoculation on the resting phenotype of our cells using both activation marker expression and a glucose uptake assay since resting cells are known to consume less glucose than activated cells [30]. We found similar levels of activation marker expression (data not shown and [6]) as well as similar levels of glucose uptake in resting cells infected with and without spinoculation (data not shown). Therefore, spinoculating resting cells is a viable model system that enhances infection levels without affecting the susceptibility of the cells to HIV integration. Overall, our results suggest that the step of integration is not restricted in resting cells.

### Infected resting CD4+T cells can produce HIV Gag (Figure 2)

**Figure 2:**
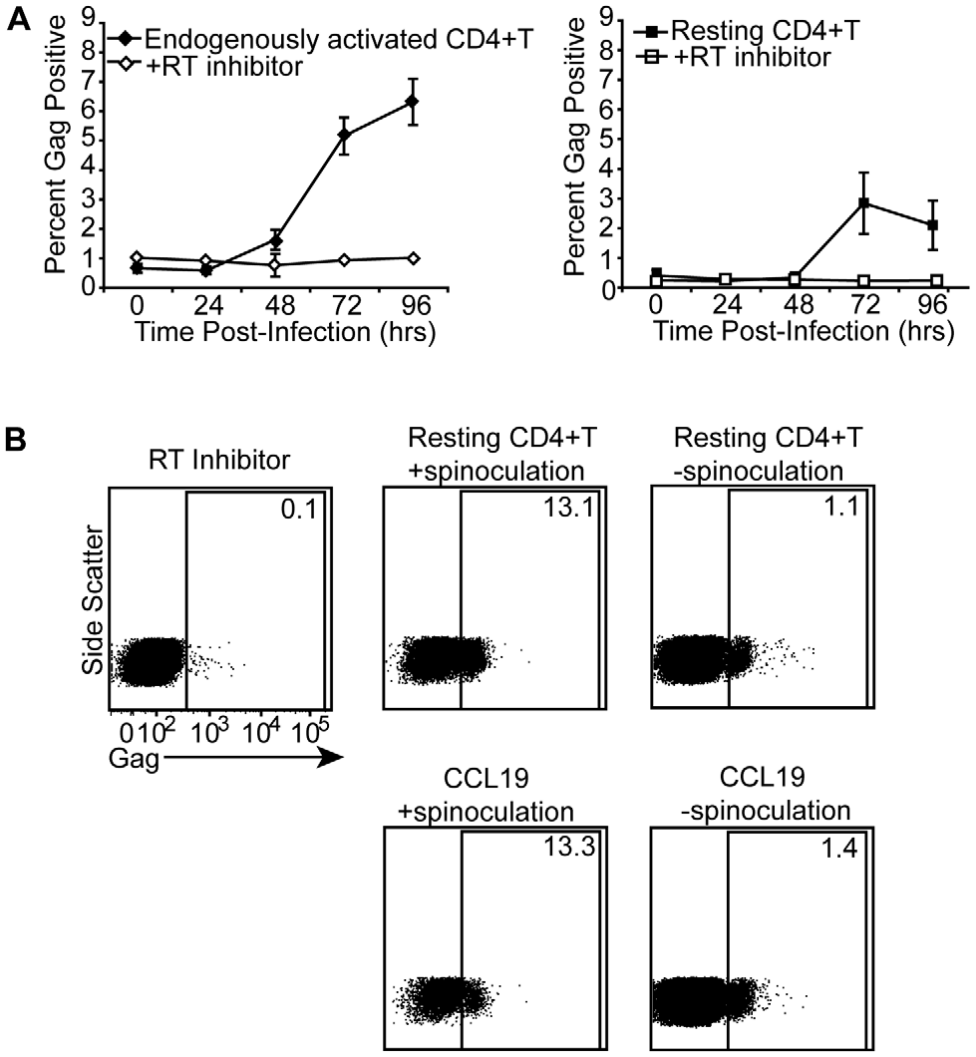
Infected resting CD4+T cells can produce HIV Gag. Bulk unstimulated CD4+T cells were spinoculated and cultured in the presence of the protease inhibitor, saquinavir. Gag protein was measured in the endogenously activated (HLA-DR+, CD25+, or CD69+) and resting (HLA-DR−,CD25−,and CD69−) cells based on activation marker expression at 0, 24, 48, 72, and 96 hours post infection. Control cells were treated with the reverse transcriptase (RT) inhibitor, efavirenz, to establish background protein levels. An average of 3 experiments in 3 different donors is shown (A). In B, purified resting or CCL19 treated cells were infected with HIV with or without spinoculation at 1200×g. Controls and gates were made as in A. Cells were gated on the activation marker negative (HLA-DR−,CD25−,CD69−) population. Approximately 10,000 events were collected for spinoculated samples while approximately 100,000 events were collected for cells that were not spinoculated. Data is a representative of 2 experiments in 2 different donors.

We next asked whether HIV infected resting CD4+T cells could produce viral proteins. Previous data has shown that phenotypically resting CD4+T cells can express SIV Gag *in vivo* in certain tissues [25], [26]. However, these results could not be repeated *in vitro* by directly infecting resting cells unless the cells were cultured in a lymphoid tissue microenvironment [18], [31]. This led to the prevailing belief that protein expression in resting cells was due to prior activation or exposure to a certain cytokine milieu. In fact, it was assumed that these Gag expressing resting cells found *in vivo* were actually productively infected and not latent [25]. However, Gag production does not necessarily mean that productive infection occurs as other viral proteins are required to make infectious particles.

To determine whether directly infected resting cells could produce Gag, we employed our spinoculation model to achieve a higher level of infection that would allow for easier detection of viral protein expression. We therefore spinoculated bulk unstimulated CD4+T cells, comprised of endogenously activated cells that expressed activation markers (HLA-DR, CD25, or CD69), and resting CD4+T cells that did not express any of these markers. We measured intracellular Gag production over time in both of these populations based on activation marker expression. We found that both resting and endogenously activated cells were capable of producing Gag above background levels (Figure 2A). However, these two populations did have slightly different kinetics (Figure 2A) consistent with our integration data (Figure 1B). We found Gag was expressed more rapidly in endogenously activated compared to resting cells (Figure 2A). However, integration levels were similar between the two populations (data not shown) indicating the cells were similarly susceptible to HIV integration.

We next examined whether these results were an artifact of spinoculation and if they could be repeated using another latency model. We therefore infected purified resting and CCL19 treated cells with or without spinoculation and monitored intracellular Gag expression. We detected Gag expressing resting cells in both latency models, with and without spinoculation (Figure 2B). Differences in Gag expression between cells infected with and without spinoculation reflected differences in integration levels (data not shown). This data suggests viral protein expression was not enhanced by spinoculation and indicates that spinoculation primarily increases viral binding (Figure 1).

We also examined which resting CD4+T cell subsets were capable of expressing Gag. To do this we sorted resting naïve, central memory, and effector memory CD4+T cells and infected them with HIV. Each subset was capable of Gag expression (Figure S1). These results indicate that resting CD4+T cells can produce Gag without stimulation in multiple CD4+T cell subsets.

### Gag expressing resting CD4+T cells remain in a latent state (Figure 3)

**Figure 3:**
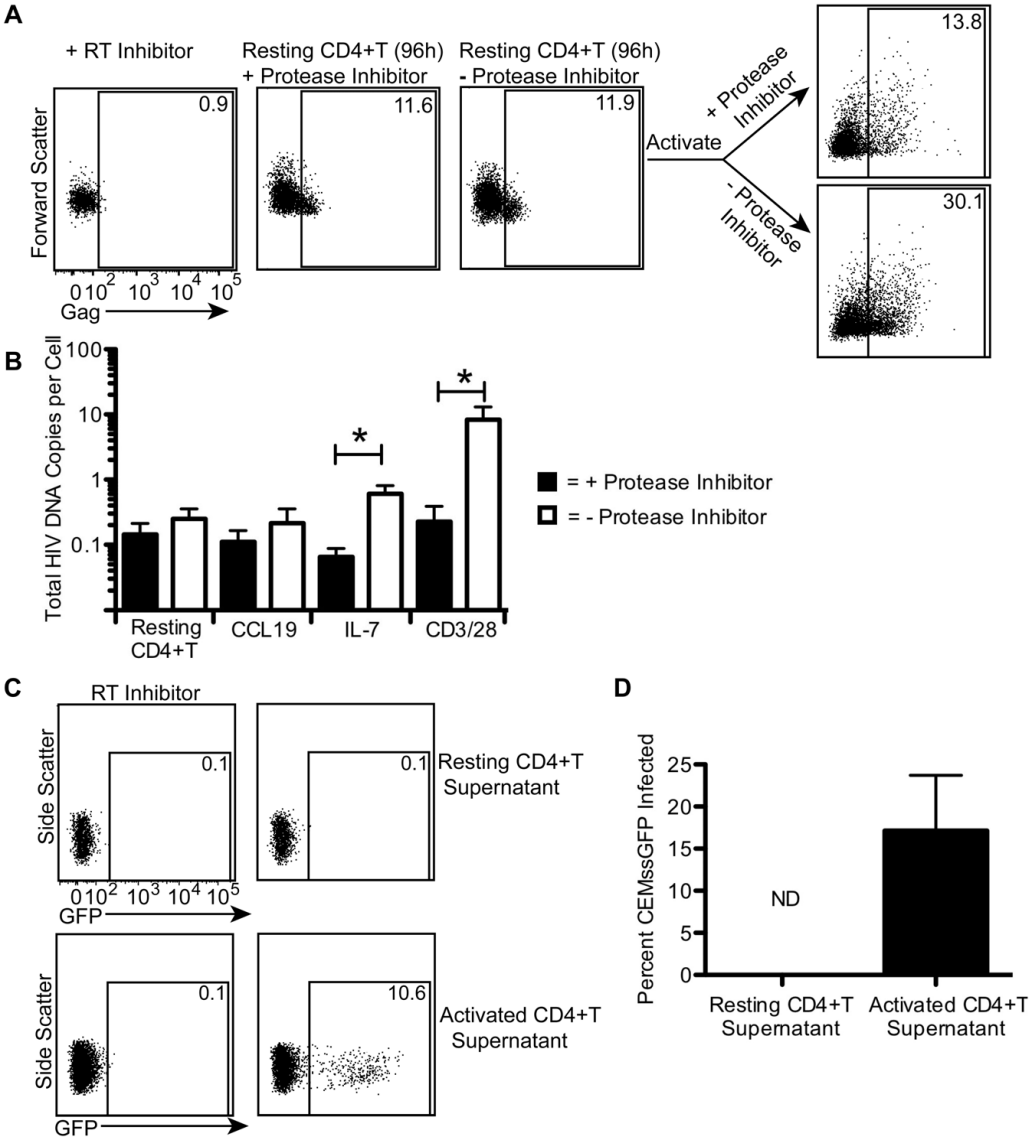
Gag expressing resting CD4+T cells remain in a latent state. In A, purified resting CD4+T cells were spinoculated with NL4-3 (MOI of 3) and cultured in the presence or absence of saquinavir for 4 days. Intracellular Gag was measured 96 hours post infection. Control cells were treated as in A and gates were set using an efavirenz treated control. Then resting cells cultured without saquinavir were stimulated with PHA+100 U/mL IL-2 for 48 hours in the presence or absence of saquinavir. Intracellular Gag was again measured. In B, cells were purified and treated as in Figure 1 but infected at a MOI of 0.2. After infection, half the cells were treated with saquinavir. Cells were collected at 72 hours for CD3/28 activated cells and at 7 days post infection for all other cells. Total DNA was measured in both fractions of cells. The average of 3 experiments with 3 different donors is shown. In C, resting and CD3/28 activated cells were spinoculated with HIV (MOI of 3). Supernatant was collected 96 hours post infection. CEMss-GFP cells were spinoculated with the collected supernatant from resting and activated cells. An efavirenz control was used to determine background GFP levels. A representative experiment is shown in C. An average of 2 experiments in 2 different donors is shown in D. Error bars represent the standard error of the measurements. *Statistically significant at p<0.05 level. ND = not detectable.

We next questioned whether the Gag production in resting cells resulted in spreading infection. To test this, we infected purified resting CD4+T cells with HIV, cultured them for 4 days in the presence or absence of a protease inhibitor, which would prevent viral spread, and then stained the cells for intracellular Gag. If the cells were latent, no spreading infection should occur and there would be no difference between cells treated or not treated with the protease inhibitor. We found the amount of Gag was similar in the samples treated with or without a protease inhibitor 96 hours post infection (Figure 3A). Similar results were achieved up to 7 days post infection (data not shown). These data indicate that spreading infection does not occur in resting cells (at least to detectable levels). We confirmed that these resting cells were latently infected by stimulating out infectious virus with phytohemagglutinin (PHA) in the presence or absence of a protease inhibitor. We found more Gag positive cells when cells were activated for 48 hours in the absence of the protease inhibitor (Figure 3A), which suggests that infectious virus was released from these cells and resulted in spreading infection. Thus, the Gag producing resting cells were latently infected.

We wanted to confirm these results were not an artifact of a high viral inoculum so we infected cells with 15-fold less virus. Since protein differences would be harder to detect at this lower MOI, we chose to use a more sensitive PCR based assay to detect spreading infection. In addition, we wanted to test the ability of different cytokines to stimulate virus production. Therefore, we compared total DNA differences between cells treated with or without a protease inhibitor. If spreading infection were to occur, we would expect to see higher levels of total HIV DNA in the cells not treated with the protease inhibitor. First, we spinoculated resting and stimulated CD4+T cells (CCL19, IL-7, or CD3/28) as in Figure 1. We then cultured the cells in the presence or absence of a protease inhibitor and measured total HIV DNA in these fractions after 7 days of culture post infection (except for CD3/28 stimulated cells, which were collected 72 hours post infection). We found that resting CD4+T cells and CCL19 treated cells had the same levels of HIV DNA in the presence or absence of a protease inhibitor (Figure 3B), indicating these cells do not produce detectable infectious virus. On the other hand, cells treated with IL-7 or CD3/28 beads showed a statistically significant increase in HIV DNA without a protease inhibitor (Figure 3B). These data suggest that resting and CCL19 treated CD4+ T cells are latently infected while IL-7 and CD3/28 treated cells are capable of supporting efficient viral spread.

As resting cells are less susceptible to infection than CD3/28 stimulated cells, it was possible that the difference in spreading infection between resting and activated cells was due to this difference in susceptibility. We therefore tested for the presence of infectious virus in the supernatant of these two cell types. We infected resting CD4+T and CD3/28 activated cells with a high inoculum as in Figure 2. We then used the supernatant of both of these cells (collected at 72 hours, 96 hours, and 120 hours post infection) to infect (via spinoculation) the activated T cell line CEMss-GFP, which expresses GFP upon HIV infection [32]. No GFP expression was detected above background when using resting CD4+T cell supernatant from any time point, but GFP was detected when using supernatant from activated cells (Figure 3C and D). Thus, we were unable to detect release of infectious virus by resting cells with three complementary methods. Overall, our results indicate latently infected cells can produce Gag without producing detectable infectious virions. We therefore wanted to determine why these cells were not productively infected.

### Resting CD4+T cells produce Gag but barely detectable levels of Env (Figure 4)

**Figure 4:**
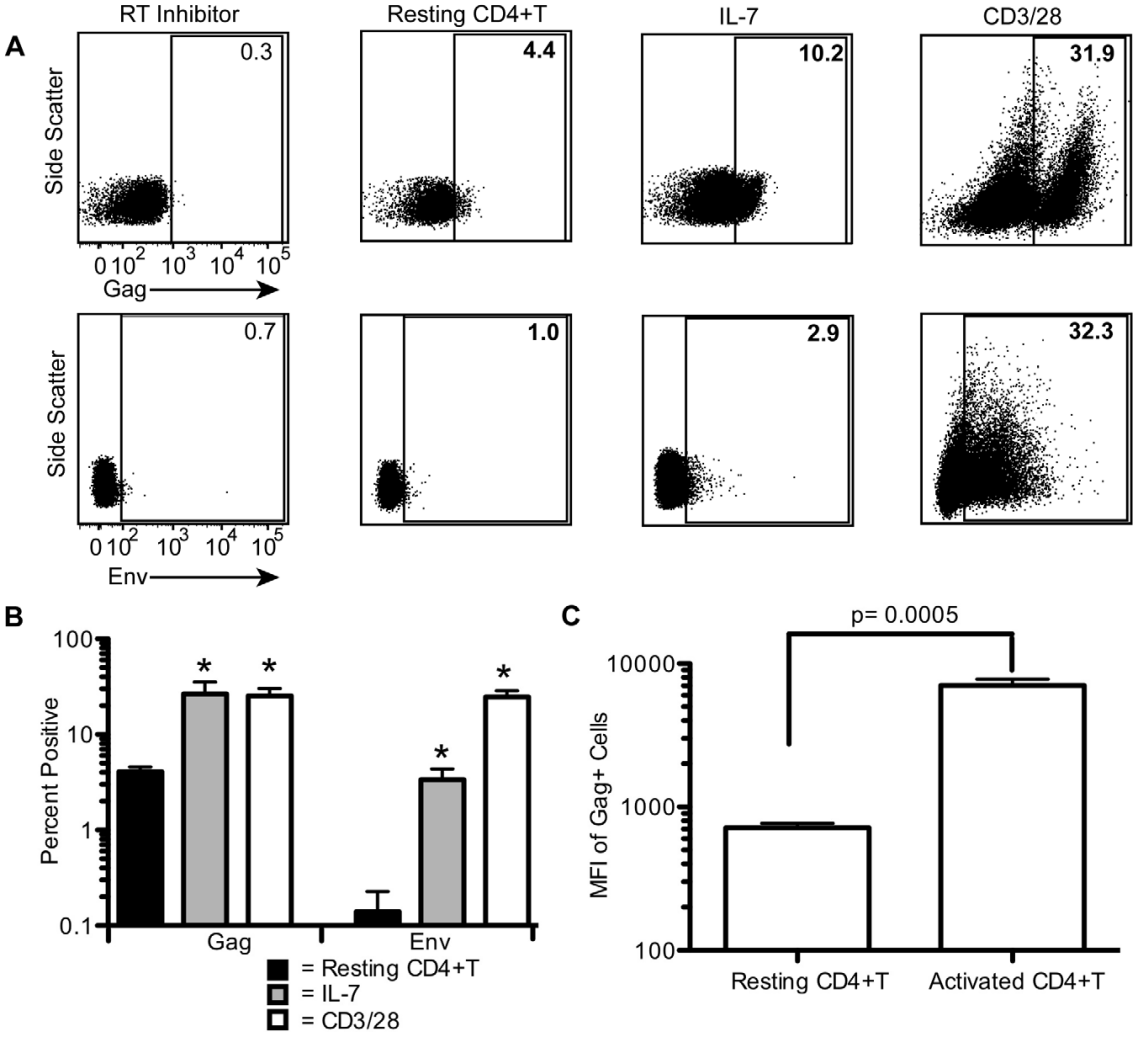
Resting CD4+T cells produce Gag but barely detectable levels of Env Cells were infected as in Figure 1 (MOI of 3) in the presence of 8 µg/mL polybrene. Cells were cultured in the presence of 25 µg/mL anti-CD4 clone 19, and Gag and Env protein levels were measured at 72 hours (48 hours in CD3/28 treated cells). A representative experiment is shown in A. An efavirenz control was used to control for background levels. The average of 3 experiments with 3 different donors is shown in B. The average MFI (median fluorescence intensity) of the Gag+ cells in resting and CD3/28 activated cells is shown in C. Error bars represent the standard error of the measurements. *Statistically significant at p<0.05 level.

As Gag, Pol, and Env proteins are absolutely required for spreading infection and we saw Gag production (and thus likely Pol) in resting cells, we next questioned whether HIV envelope could be detected in these cells. To test this, we spinoculated purified resting cells and measured Env expression on the surface of these cells. Interestingly, resting cells produced little to no detectable Env protein above background levels (Figure 4 A and B, Env positive cells were undetectable in 1 of 3 experiments and barely detectable in 2 of 3 experiments), even though infection levels reached ∼70% based on integration levels (data not shown). IL-7 and CD3/28 treated cells, on the other hand, consistently led to detectable and significantly higher levels of Env positive cells (3.4% and 24.6%; p = 0.015 and 0.0018 respectively, Figure 4B). Thus, infected cells capable of spreading infection were more frequently positive for Env. It is possible that resting cells may produce low levels of Env that might be sufficient for low levels of spreading infection. However, if viral spread occurs in resting cells, it occurs at very low levels not detectable with our assays.

We then examined Gag production as a control to see if IL-7 and CD3/28 had similar enhancements on Gag expression, or if enhanced translation was limited to Env. We found on average 4.1% of resting cells were positive for HIV Gag (Figure 4B) while IL-7 and CD3/28 treatment led to statistically higher levels of Gag positive cells (p = 0.032 and 0.0059, respectively), indicating enhanced protein expression was not limited to Env. We found that CD3/28 activated cells had not only a ∼10 fold higher frequency of Gag expression than resting cells but also ∼10 fold higher levels of Gag expression (MFI, Figure 4C, p = 0.0005). These data are consistent with studies *in vivo* suggesting resting cells release produce approximately 10 fold less Gag than activated cells during SIV infection [26]. Our results suggest that stimulation may globally upregulate HIV protein expression and not just increase one particular viral protein.

### Protein expression differences are reflected at the transcript level (Figure 5)

**Figure 5:**
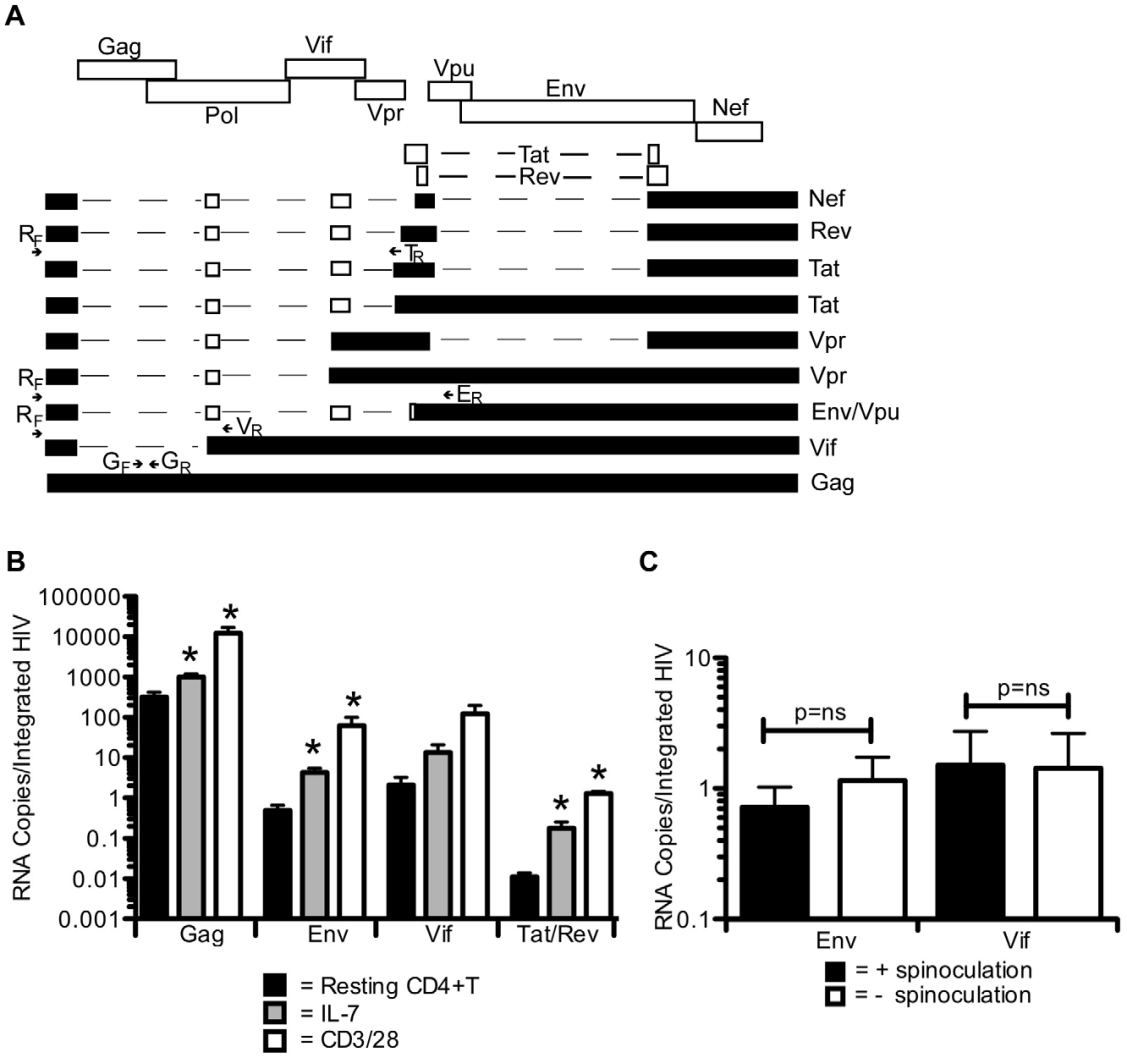
Protein expression differences are reflected at the transcript level. A schematic showing the different HIV RNA species and the primers used to detect them is shown in A. The upper portion depicts the viral genome while the lower portion depicts the various RNA transcripts. For the different RNA species, black boxes represent regions present in all forms of the indicated viral transcript while white boxes represent regions that may or may not be present. G_F_ and G_R_ were used to detect *gag* levels. R_F_ and E_R_ were used to detect *env*. R_F_ and V_R_ were used to detect *vif*, and R_F_ and T_R_ were used to detect *tat/rev*. Primers were confirmed to solely detect their respective products by gel electrophoresis. Cells were treated and infected as in Figure 4. Cells were cultured in the presence or absence of the integrase inhibitor, raltegravir. RNA was collected at 72 hours (48 hours in CD3/28 treated cells). RNA levels were calculated per cell and were normalized to integration levels. *gag* levels were obtained by subtracting the levels of *gag*/cell in the raltegravir treated fraction from the levels in the - raltegravir fraction. An average of 3 experiments in 3 different donors is shown (B). In C, resting cells were infected with or without spinoculation and *env* and *vif* levels were measured as in B. An average of 3 experiments in 3 different donors is shown. Error bars represent the standard error of the measurements. *Statistically different at the p<0.05 level.

We next examined if the differences in protein levels were due to differences in transcription and/or splicing. We first measured the levels of *gag* RNA to compare with our protein measurements. Because virions contain *gag* RNA, we needed to compensate for background RNA from incoming virions as described in the Methods. We calculated the amount of unspliced RNA in resting cells to be 300 copies per integrated HIV (Figure 5, S3). There were significantly higher levels of *gag* in IL-7 and CD3/28 treated cells (1000 and 12000 copies per integrated HIV, p = 0.015 and p = 0.032 respectively), which corresponds with the protein levels in Figure 4 and are consistent with the different RNA levels detected *in vivo* [25].

We next tested if levels of envelope protein also corresponded with the amount of *env* transcripts in resting cells. We measured *env* levels using the primers depicted in Figure 5A. Resting cells contained 0.5 copies per integrated HIV while IL-7 treated cells produced approximately 10 fold more *env* RNA and CD3/28 treated cells produced roughly 130 fold more *env* RNA (Figure 5B, p = 0.017 and 0.088 respectively). These results indicate that Env protein levels matched *env* RNA levels, suggesting a pre-translation block in resting CD4+T cells.

We next tested whether other spliced transcripts were also low in resting cells. We began by measuring levels of *vif* since it is singly spliced and the only transcript that encodes the Vif protein. *Vif* RNA levels followed the same pattern as *gag* and *env* with both IL-7 and CD3/28 treated cells trending to produce more RNA (approximately 6 and 60 fold) than resting cells (2.1 copies/integrated HIV), (Figure 5B, p = 0.107, p = 0.084 respectively). These results suggest that the effects of stimulation were similar among various splice products and that a specific block to certain spliced forms did not explain the lack of productive infection in resting cells.

We then simultaneously quantified both *tat* and *rev* transcripts, designated *tat/rev*. Tat is the major transcriptional regulator of HIV while Rev promotes RNA export out of the nucleus. *Tat/rev* RNA was barely detectable in resting cells (0.01 copies per integrated HIV, Figure 5B). IL-7 and CD3/28 treatment resulted in significantly more *tat/rev* transcripts (20 and 130-fold more, p = 0.043 and p = 0.0005 respectively). Thus, it is possible that these higher *tat/rev* levels could explain transcriptional differences between resting and stimulated cells.

Since it remained possible that spinoculation artificially enhanced HIV RNA expression, we repeated our RNA measurements in resting cells infected with and without spinoculation (Figure 5C). We quantified *env* and *vif* expression as their quantitation is more robust than *gag* since there is no contribution from incoming virus. We found similar levels of both *env* and *vif* per integrated HIV in cells infected with or without spinoculation (Figure 5C). Thus, our viral transcription data is not an artifact of spinoculation.

Overall, our RNA data indicate that while substantial levels of unspliced transcripts exist in resting CD4+T cells, there are significantly less spliced messages formed in these cells. This suggests that resting cells may not have sufficient levels of spliced products required for spreading infection, therefore providing a potential mechanism for the absence of viral spread in these cells. Enhanced transcription in activated cells not only increases the level of *gag* transcripts, but also increases viral spliced products to a level that allows efficient spread.

### Differences in HIV integration site selection between Gag positive and negative cells (Figure 6)

**Figure 6:**
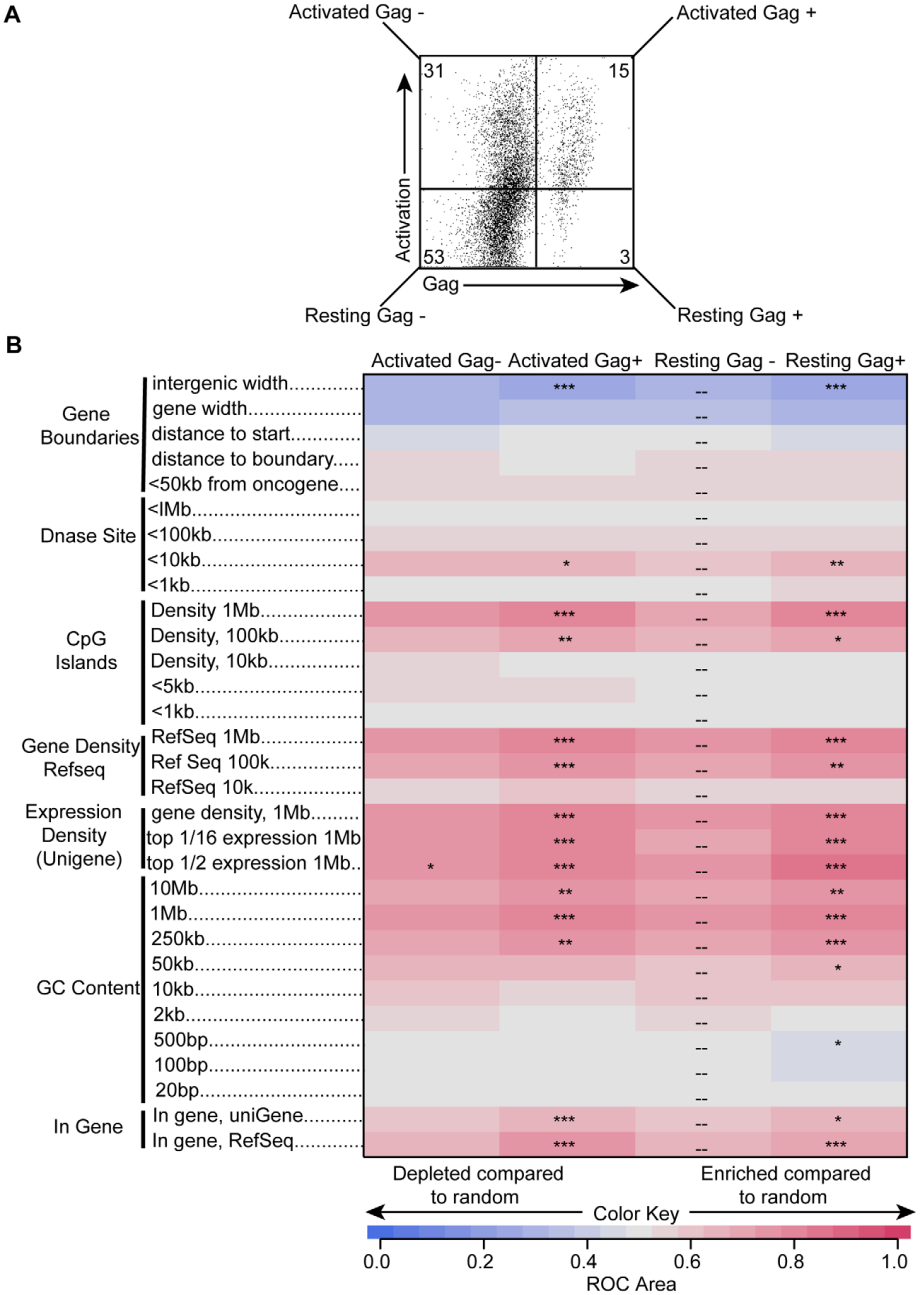
Differences in HIV integration site selection between Gag positive and negative cells. Unstimulated cells were spinoculated with NL4-3. At 96 hours post infection, cells were stained with antibodies for the activation markers HLA-DR, CD25, and CD69 as well as for intracellular Gag. Cells were sorted into 4 populations: activated Gag−, activated Gag+, resting Gag−, and resting Gag+. Numbers reflect the percentage of each population in the total infected unstimulated cells (A). A heatmap showing relationships of integration site distributions to various genomic features is shown in (B). Each column represents a different population and each row a genomic feature. Darker shades of red indicate associations observed more than random and darker shades of blue indicate less than random. *p<0.05, **p<0.01, ***p<0.001.

Since we detected viral protein production in a population of resting cells, we asked why some cells produced viral protein and others did not. As integration site selection could explain a difference in protein production, we tested whether HIV integration site selection was different in Gag positive and negative cells. To do this, we sorted infected CD4+T cells into 4 populations based on activation status (resting and activated) and Gag expression (Gag positive and Gag negative), confirmed infection in each subset using HIV DNA measurements, and analyzed where in the human genome HIV integrated in each of these populations (Figure 6A). We found statistically significant differences between Gag positive and Gag negative resting cells (Figure 6B), reaffirming that these are two distinct populations. Surprisingly, the differences were similar to those found between Gag positive and negative activated cells (Figure 6B). Our data indicate that there were greater similarities in integration site selection among any Gag producing cells than among cells with the same activation state. This suggests that differences in integration site selection were in fact reflective of viral protein production. However, these differences were small. There was also a modest increase in integration frequency near heterochromatic alphoid repeats in the Gag negative subsets (data not shown), paralleling previous studies [33], [34]. Nevertheless, even in cells not expressing viral proteins, HIV still preferred to integrate in active genes, just to a lesser extent than in Gag producing cells. This pattern was also seen for other genomic features associated with active genes such as GC content and CpG islands (Figure 6, [35]). Overall, the differences in integration site selection explain a small part of the differences between Gag positive and negative cells.

## Discussion

We have previously shown that resting CD4+T cells are susceptible to HIV integration without stimulation [6]. Here, we employed a latency model that achieves high levels of infection without requiring stimulation to identify a population of latently infected resting CD4+T cells that express Gag but do not support viral spread. The block to productive infection corresponded with barely detectable levels of envelope protein, potentially due to low levels of *tat* in these cells. Thus, our latent model reveals that there is a continuum of latent cells from cells in a pre-integration state of latency (not focused on in this report) to translationally silent cells to cells expressing HIV proteins without spreading infection. The ability of some latent cells to produce viral proteins has important implications for therapies targeting the latent reservoir as these cells could be recognized by a robust immune response.

Previous work has shown that phenotypically resting cells can produce HIV and SIV RNA and protein *in vivo* in certain tissues [25], [26]. However, it is impossible for *in vivo* studies to determine at what activation state the resting cells are actually infected, whether the *in vivo* cytokine milieu is required for the expression of viral proteins, and whether the cells are productively infected. Here we show by RT-PCR and flow cytometry that directly infected resting cells can produce HIV Gag *in vitro* without additional stimulation while remaining latently infected. Our results also reveal that CCL19 does not enhance integration efficiency (Figure 1), as has been shown recently [10]. This discrepancy is not due to spinoculation or to cytokine concentration as we repeated our results without spinoculation using several doses of CCL19, up to 1 µM, and confirmed the activity of our cytokine preparation in a chemotaxis assay (data not shown). It is possible that differences in sera used, potency of the virus, or the sensitivity of our integration assay could explain this difference.

We show that the majority of resting Gag positive cells are latent in that they are unable to support productive infection based on similar protein and DNA measurements in protease treated and untreated fractions (Figure 3 A,B). Additionally, the supernatant of infected resting cells cannot infect CEMss-GFP cells (Figure 3C, D). However, these cells can release infectious virus upon activation (Figure 3A). The block to productive infection appears to be due, at least in part, to the barely detectable levels of envelope protein in these cells (Figure 4). IL-7 and CD3/28 treatments were able to overcome this block and resulted in both higher levels of envelope protein and spreading infection. Our results are consistent with the extensive evidence that resting cells are not capable of productive infection in the absence of various stimuli (reviewed in [15]). While it is conceivable that a small number of resting cells are able to release infectious virions in our model, these cells are so few that we could not detect viral spread. Additionally, as our cultures can never be 100% pure, it is almost impossible to conclusively prove that any low level spreading infection results from resting cells instead of contaminating activated CD4+T cells. Nonetheless, it is clear that infected resting cells are very inefficient at viral spread and this may be the most important difference between HIV infection in resting and activated CD4+T cells.

Although we found that resting CD4+T cells in our model do not support productive infection, calling these cells latent may still seem controversial. Several authors claim that any cells producing viral proteins are not latent since protein production will result in elimination of the cell due to cytotoxic effects or immune clearance [34], [36]. With regards to cytotoxicity, the Gag positive resting cells described here produce both quantitatively less and fewer types of viral proteins than activated cells (Figure 4) and so may be better able to survive any cytotoxic effects of the viral proteins. Notably, Env is particularly known for its toxicity and so low Env expression should enhance the longevity of resting cells [37], [38]. Additionally, the longer half-lives of resting cells may allow them to survive better in the face of protein production than their activated counterparts [39]. Furthermore, recent work has shown that latently infected cells from patients on HAART produced Gag when treated with SAHA but were not killed by HIV cytopathic effects and had substantially longer half-lives than expected [40]. With regards to immune clearance, since resting cells are producing less protein than activated cells, they may be harder for the immune system to clear. Just because a cell may be an immune target does not mean it will be successfully cleared, especially since the immune system declines over the course of HIV infection [41] and the frequency of HIV-specific CD8+T cells decreases on HAART [42], [43]. A recent study showed that while CD8+T cells from elite suppressors (ES) could clear latent cells stimulated to produce HIV Gag *in vitro*, the same was not true for patients on HAART [40]. Overall, while some Gag positive resting cells may die due to cytotoxicity or to immune clearance, it is likely that a subset of these cells will survive. This is consistent with the fact that resting cells containing viral RNA are detected on HAART [19], [44]. Since the cells do not produce infectious virions and likely persist on HAART, they should be considered latent.

As we found Gag but little Env production in resting cells, the obvious question was whether this difference was due to blocks at transcription, splicing, and/or other post-transcriptional steps. We found that the regulation of protein expression was mainly at the level of viral transcripts (Figure 5). *gag* RNA levels were substantially higher than that of any spliced transcript in resting cells, suggesting splicing frequency may be low in resting cells; however, stimulation enhanced the amounts of *gag*, *env*, *vif*, and *tat/rev* (unspliced and spliced RNA species) to similar extents (Figure 5). Our *gag* levels were similar to those described *in* vivo [25]. These data show that activation globally enhances levels of all viral transcripts, suggesting there is no specific block to any particular spliced form in resting cells. Instead, each splice product was likely controlled by the strength of the splice site, allowing higher levels of some transcripts over others. For example, it is known that the *tat* splice site is relatively weak, which may explain the low levels of *tat* in resting cells [45].

The higher levels of transcription in activated cells corresponded with an increase in *tat/rev* RNA levels, which were barely detectable in resting cells (Figure 5). Transcriptional efficiency in resting cells was thus not likely due to Tat but to other transcription factors. NFAT and NF-κB are important regulators of HIV transcription and are known to have low activity in resting cells [15], [46]. But how then do we explain any transcription in these cells much less protein expression? First, it is possible that other transcription factors, such as TCF, may be involved in HIV transcription and translation in quiescent cells [47], [48]. Second, it is possible that transcriptional activity from surrounding genes could explain HIV transcription in certain cells, particularly if the HIV provirus is integrated in a transcription unit in the same orientation as the host gene [33], [46], [49]. This would suggest integration site selection would play a role in Gag expression. Indeed, we found that there were small but statistically significant differences in integration site selection between Gag expressing and non-expressing resting cells (Figure 6). However, the differences were small and unlikely to fully explain differences in viral protein expression.

A related question is how Gag expression occurs without Rev, which plays a critical role in nuclear export of unspliced and singly spliced HIV RNA (reviewed in [50]). First, it is possible that low levels of Rev are expressed in resting cells allowing successful nuclear export of *gag*; however, the low levels of *rev* RNA makes this unlikely. Second, it is possible that due to low levels of HIV splicing in resting cells (Figure 5), enough *gag* RNA accumulates to allow inefficient export into the cytoplasm. As we see substantial *gag* levels in resting cells (∼300 copies of *gag*/integrated HIV), there may be sufficient quantities of unspliced RNA to enable some RNA to exit the nucleus. Our data is consistent with published data showing higher levels of unspliced than spliced RNA in resting CD4+T cells and PBMC in patients on HAART [44], [51]–[53]. If HIV splicing were to occur efficiently in resting cells, one would expect spliced forms would be more prevalent than unspliced RNA due to the low levels of *rev* (Figure 5 and [52]). Additionally, our data is consistent with data from Zack and colleagues [54] showing that while spliced forms are detected earlier in pre-stimulated HIV infected cells, latent cells that are activated produce detectable *gag* RNA before spliced forms.

Overall, our RNA data agree with prior literature, as described above. Furthermore, our data are likely consistent with a described block to nuclear export of HIV RNA in resting cells [19], [22] as we see the expected pattern of high *gag* RNA levels but a substantially lower percentage of Gag positive cells (∼4%, Figure 4). Nonetheless, the export block was not absolute as proteins were translated, indicating a fraction of RNA is transported to the cytoplasm. While sufficient unspliced RNA must exist in resting cells to result in nuclear export and translation of Gag, the same could not be said for Env. In fact, a nuclear export block is consistent with our data that we do not see substantial Env expression even though there are approximately 0.5 *env* copies per integrated HIV DNA (Figure 5). Our model therefore suggests the block to productive resting cell infection is not absolute but is instead a series of less efficient steps that result in significantly different outcomes in resting and activated cells. Thus, resting cell infection mainly results in latent infection while infection in activated cells primarily results in productive infection.

Our spinoculation model generated higher levels of infection than typically obtained by other models (e.g. 70% of cells contained integrated HIV). This high frequency of infection was essential to demonstrate HIV proteins were expressed in a subset of latently infected cells. However, as cells are not spinoculated *in vivo*, we needed to confirm that spinoculation did not artificially affect our results, as has recently been suggested [27]. While our prior studies indicated that the major effect of spinoculation was at the level of binding [55], we show here that spinoculation did not enhance integration efficiency (Figure 1C,D) or transcriptional efficiency (Figure 5C) in resting cells, consistent with our prior reports showing similar efficiencies of integration per bound virion with or without spinoculation, even at low viral inoculums [24]. As previously mentioned, our RNA and protein data are also consistent with *in vivo* results (Figure 2, 4 and 5, [19], [25], [26], [44], [51]). Furthermore, our data were not the result of an artificially high viral inoculum as similar outcomes of infection were seen with or without spinoculation at the level of integration (Figure 1), transcription (Figure 5C), translation (Figure 2B) and viral spread (Figure 3B). While signaling may occur due to spinoculation and the physiology of the cells may be altered, our data show that these changes do not affect the course or efficiency of HIV infection in these cells. Overall, our data suggests that spinoculation is a valid and useful model for studying HIV latency as it only affects the frequency of infected cells that progress through the HIV life cycle rather than the course of infection.

The ability of latently infected resting cells to express Gag protein without spreading infection has important implications for treating the latent reservoir. First, as some latently infected resting cells can produce protein, they may be cleared by the immune system, particularly in patients with strong immune responses to HIV such as ES [56], [57]. This could potentially explain the lower reservoir levels in these patients [58]. Our data suggest that developing strategies to boost the immune response in patients could be vital in clearing the latent reservoir. Second, since resting cells can produce Gag protein but much less Env, strategies targeting Gag producing cells may have added benefits compared to therapies targeting Env expressing cells. Currently, several studies have begun targeting Env producing cells through immunotoxin approaches (reviewed in [59]). Since resting cells do not produce substantial Env levels, these types of therapy would be unable to target latent resting CD4+T cells without stimulation; however, therapies targeting Gag may be able to eliminate some latent cells. Finally, our data indicate that resting cells can produce protein without spreading infection. Thus it may be possible to develop therapies that will stimulate cells to produce enough protein to be cleared without causing ongoing replication.

Targeting and eliminating the latent reservoir has become an attractive approach for curing HIV. Here we describe a direct infection *in vitro* latency model that allows sufficient infection levels for study without requiring artificial stimulation and the related physiological consequences on the cells. Using this model, we show resting cells are capable of producing Gag protein without spreading infection. Our model thus contains an extensive range of latently infected resting CD4+T cells from cells in a pre-integration latent state to cells producing protein without spreading infection. By including all of these populations, our model may better represent HIV latency *in vivo*. Through the use of spinoculation, we can generate sufficient levels of infected cells to study the differences between latent cells capable of Gag production and cells incapable of protein expression. Characterizing these differences may lead to therapies that could turn translationally silent cells into Gag expressing latent cells that could be cleared by a robust immune response.

## Methods

### Ethics statement

Primary human CD4+T cells used in these studies were obtained through anonymous donation to the University of Pennsylvania's Center for AIDS Research Human Immunology Core after written informed consent and approval by the University of Pennsylvania's institutional review board.

### Isolation of resting CD4+T cells

Unstimulated CD4+T cells were purified from leukapheresis-enriched PBMC using Rosette Sep (Stemcell Technologies) and were obtained from the University of Pennsylvania's Center for AIDS Research Human Immunology Core. To purify resting CD4+T cells, cells were stained with PE labeled antibodies against CD25, CD69, and HLA-DR (BD Biosciences) and anti-PE magnetic beads (Miltenyi Biotec) as recommended by the manufacturer. Cells were then depleted using LD columns as recommended by the manufacturer (Miltenyi Biotec). Resting CD4+T cells were typically greater than 98% pure.

### Cell stimulation and infection

Resting CD4+T cells were either left untreated in RPMI containing 10% heat inactivated FCS (Invitrogen, Qualified) supplemented with 100 µg/mL penicillin-streptomycin and GlutaMax (Invitrogen), or were stimulated with 20 ng/mL IL-7, 100 nM CCL19 (R&D Biosystems) or with CD3/28 beads at a concentration of 3 beads/cell (Invitrogen) with 100 U/mL IL-2 (R&D Biosystems) for three days. Cells were then spinoculated at a concentration of 1×10^7^ cells/mL in viral transfection supernatant (MOI of 3 as assessed by infection of CEMss-GFP cells, unless otherwise noted) for 2 hours at 1,200×g at 25°C (unless otherwise indicated). NL4-3 viral stocks were prepared by 293T transfections by the University of Pennsylvania's Center for AIDS Research Viral/Molecular Core. After spinoculation, cells were washed twice in CO_2_ independent media (Invitrogen) and treated with 50 µg/mL *Dnase* I (Roche) and 10 mM MgCL_2_ to remove plasmid DNA. Cells were then cultured in the presence of 1.25 µM of the protease inhibitor saquinavir (Roche) to prevent viral spread (unless otherwise noted). For experiments measuring Env protein and RNA, cells were infected in the presence of 8 µg/mL polybrene (Millipore), excluding infections of resting cells without spinoculation.

### Viral binding

Viral binding was estimated by measuring cell-associated p24 via a p24-specific enzyme-linked immunosorbent assay (Perkin-Elmer) as previously described [23].

### Reverse transcription and integration

DNA was prepared after infection using the QIAamp DNA Micro Kit (Qiagen). Real-time PCR was used to detect total HIV DNA (complete SST), β-globin, and integrated DNA as previously described [24].

### RNA isolation

RNA was isolated using Tri-Reagent (Sigma Aldritch) as per the manufacturer's recommendations in the presence of 10 µg/mL Glycogen (Roche). Two chloroform extractions were performed and 75% of the RNA fraction was collected both times to ensure RNA purity and to achieve a predictable yield. Cells were counted prior to isolation using the Countess (Invitrogen) automated cell counter. Using the predicted percent RNA yield described above, cell concentrations were calculated. These counts were independently confirmed by 18S RNA measurements based on cell type (resting CD4+T cells, CD3/28 activated cells etc). RNA copies were quantified per cell based on these calculations.

### Reverse-transcriptase PCR standard generation

Gag standards were generated as previously described [55]. Standards for *vif*, *env* and *tat/rev* were all generated in the following way. Reverse primers, as explained in Figure 4A, for *vif*, *env* or *tat/rev* were used to generate cDNA from RNA isolated from our CEMss integration standard cell line [60]. To generate cDNA, 200 ng of total RNA was added to a reaction with the reverse primer only in a master mix following the High Capacity cDNA Reverse Transcription kit protocol (Applied Biosystems). The following cycling conditions were used: 25°C for 10 minutes, 37°C for 120 minutes and 85°C for 5 minutes. The cDNA was then diluted 1∶10 in 10 mM Tris-HCl pH 8.0 and 4 µL of this dilution was added to a PCR reaction with the following: 1× buffer (Invitrogen), 3.5 mM MgCl_2_, 300 µM dNTPs, 100 nM forward (Figure 4A) and reverse primers and H_2_O to a total volume of 20 µL. The following cycling conditions were used: 95°C for 10 minutes and 40 cycles of 95°C for 30 seconds, 55°C for 30 seconds and 72°C for 1 minute. The resulting products were run on a 2% agarose gel. Appropriate sized bands were excised and purified with the QIAquick gel extraction kit (Qiagen). This purified cDNA was then cloned into the pCR2.1 TOPO vector following the TOPO TA cloning kit (Invitrogen). Chemically competent *E. coli* cells (Invitrogen) were transformed with the plasmids and grown on LB agar (Becton Dickinson) with ampicillin (Sigma) overnight at 37°C. Colonies were selected, shaken overnight in LB broth (Becton Dickinson) with ampicillin at 37°C. Plasmids were extracted with the Qiaprep Spin Miniprep Kit (Qiagen). A digestion with *Eco*RI (New England Biolabs) was performed to confirm presence of the appropriate sized band. Clones with appropriate bands were also sequenced to verify we obtained the appropriate RNA splice forms. After sequence confirmation, appropriate clones were grown in large cultures overnight in LB broth with ampicillin at 37°C. Plasmids were isolated following the PureLink HiPure Plasmid Filter Maxiprep Kit (Invitrogen) and eluted into 500 µL of Tris-HCl pH 8.0. Confirmatory digestion and sequencing were again performed. Plasmids were then linearized by digestion with *Spe*I (New England Biolabs). The *Spe*I enzyme was heat inactivated after digestion for 20 minutes at 80°C. Plasmids were then *in vitro* transcribed to generate RNA using the HiScribe T7 *in vitro* Transcription Kit (New England Biolabs). RNA was purified using the RNeasy MinElute Cleanup kit (Qiagen) including the optional on column DNase treatment (Qiagen) to remove leftover plasmid DNA. Finally, RNA was measured by spectrophotometry and the copy numbers were calculated based on the concentration and number of bases per RNA transcript. We confirmed our primers solely detected their respective products via gel electrophoresis.

### RT-PCR

RT-PCR reactions were performed using a one-step reaction at 20 µL total volume using the High Capacity cDNA Reverse Transcription Kit (Applied Biosystems). Reactions contained 280 nM dNTP, 1.68 nM of each primer, and 0.56 nM of probe. Platinum Taq polymerase (Invitrogen) was used at 0.75 U/reaction and reverse transcriptase was used at 7.5 U/reaction (Applied Biosystems). The RT-PCR was run on an ABI 7500 Fast Instrument with the following protocol: 1) 95°C for 30 min 2) 95°C for 15 s 3) 60°C for 30 s 4) 72°C for 1 min. Steps 2–4 were repeated for 40 cycles. RT-PCR for *env*, *vif*, and *tat/rev* were all performed with the RU5 forward primer (R_F_) 5′GCCTCAATAAAGCTTGCCTTGA-3′ and the probe 5′CCAGAGTCACACAACAGACGGGCACA-3′. The reverse primer for *env* (E_R_) was 5′-GATTACTATGGACCACACAACTATTG-3′. The reverse primer for *vif* (V_F_) was 5′-CCATGTGTTAATCCTCATCCTGTC-3′. The reverse primer for *tat/rev* was 5′-CTTCTTCCTGCCATAGGAGATGCC -3′. *gag* was detected using the forward primer (G_F_) 5′-AGTTGGAGGACATCAAGCAGCCATGCAAAT-3′, the reverse primer (G_R_) 5′-YGCTATGTCAGTTCCCCTTGGTTCTCT-3′, and the probe 5′-GCGAGCGAGACCATCAATGAGGAAGCTGCAGA-3′.

### Gag background

As has recently been described [22], measuring *gag* levels in resting cells is difficult since the viral genome, like *gag*, is unspliced. Therefore, *gag* levels were calculated by subtracting the *gag*/integrated DNA signal in cells treated with 1 µM of the integrase inhibitor raltegravir (AIDS Reagent Program) from the *gag*/integrated DNA signal in uninhibited infected cells.

### Protein staining and flow cytometry

For Gag staining, cells were fixed and permeabilized using the Fix and Perm Cell Permeabilization Kit (Invitrogen) as recommended by the manufacturer and were intracellularly stained with a KC57-FITC antibody (Beckman Coulter). For HIV Env staining, cells were incubated with a gp120 clone 2G12 antibody (AIDS Reagent Program). Cells were then washed twice with PBS. Next, cells were stained with a mouse anti-human IgG PE conjugated antibody (Southern Biotech). Control cells were treated with 1 µM efavirenz (AIDS Reagent Program) post inoculation to serve as a negative control for flow cytometry gating. To ensure Env signal was a result of actual production by the cell and not release of virus and rebinding to a neighboring cell, cells were cultured in the presence of 25 µg/mL anti-CD4 clone 19 (generously donated by Ron Collman).

### Cell sorting

For integration site selection analysis, infected unstimulated CD4+T cells were stained with intracellular Gag and activation markers as described above. Cells were sorted using a FACSAria II Cell Sorter (BD Bioscience) into four populations: activated Gag− (92% pure), activated Gag+ (95% pure), resting Gag− (99% pure), resting Gag+ (97% pure). The main contaminant for each population was resting Gag− cells.

### CD4+T cell subset analysis

Uninfected PBMC were stained with FITC-conjugated lineage markers (CD8, CD11c, CD14, CD16, CD20, CD56, BDCA-2) and PE-conjugated activation markers (CD25, CD69, HLA-DR) to mark resting CD4+T cells (BD Bioscience). Cells were also labeled with CD45RO PE-Texas Red (Beckman Coulter) and CCR7 PerCpCy5.5 (BD Bioscience) to distinguish naïve (CD45RO−, CCR7+), central memory (CD45RO+, CCR7+) and effector memory cells (CD45RO+, CCR7−). These resting subsets were sorted using a FACSAria II Cell Sorter (BD Bioscience). Naïve cells were typically 99% pure while central memory and effector memory cells were typically >95% pure. Sorted subsets were then infected with NL4-3 and intracellular p24 was quantified 72 hours post infection as above.

### Integration Site Recovery and Analysis

Genomic DNA was digested overnight using *Mse*I and *Tsp*509I. Fragments from each sample were then ligated overnight at 16°C to their own unique PCR adapter. To isolate integration site junctions, two rounds of PCR were performed as previously described [61] with a set of nested primers specific for each linker and the viral LTR. Amplification products were sequenced using 454 sequencing. DNA barcodes were included in the nested LTR primers to allow sample pooling prior to sequencing [61], [62].

DNA sequences that contained an exact match to the terminal LTR sequence (TCTAGCA; lies between the LTR primer and the site of integration), aligned within three base pairs of the beginning of the sequence read, and had a single best alignment with ≥98% identity to the human genome (hg18, version 36.1) were counted as true integration events. Random genomic sites were computationally selected for comparison (matched random controls, [63]). Gene expression analysis was based on microarray data from T cells [64]. All sequences will be deposited in the SRA repository upon acceptance of this manuscript for publication.

### Statistical analysis

A one-tailed Student's t-test with the Holmes correction was used to compare statistical differences between experimental conditions (Graphpad Prism). For the spinoculation comparisons in Figure 2E, a one-way ANOVA was performed with a Bonferoni post test. Logistic regression and other statistical methods as described in [35], [63] were used to compare distributions of integration sites to those of genomic features.

## Supporting Information

Figure S1 Gag is expressed in several CD4+T cell subsets. Uninfected PBMCs were sorted into resting naïve (CCR7+, CD45RO−), central memory (CM; CCR7+, CD45RO+) and effector memory (EM; CCR7−, CD45RO+) CD4+T cells. Central and effector memory cells were typically >95% pure while naïve cells were typically 99% pure. The CD4+T cell subsets were then spinoculated with NL4-3. Gag expression was measured 72 hours post infection. An efavirenz control was used to determine background levels of Gag. A representative sort strategy is shown in A. A representative experiment is shown in B. In C, an average of 2 experiments in 2 different donors is shown.
(TIF)

Figure S2 *Gag* background. Cells were infected and treated as in Figure 4. Levels of RNA in the integrase inhibitor treated and untreated samples are shown. Data are the average of 3 experiments in 3 different donors. The calculated levels of transcribed *gag* are reported in Figure 5. *Statistically different at the p<0.05 level.
(TIF)
