# Supplementary figures and images for: Directly Infected Resting CD4+T Cells Can Produce HIV Gag without Spreading Infection in a Model of HIV Latency
Source: PLoS Pathog. 2012 Jul 26;8(7):e1002818. doi: 10.1371/journal.ppat.1002818 (PMC3406090; doi:10.1371/journal.ppat.1002818)

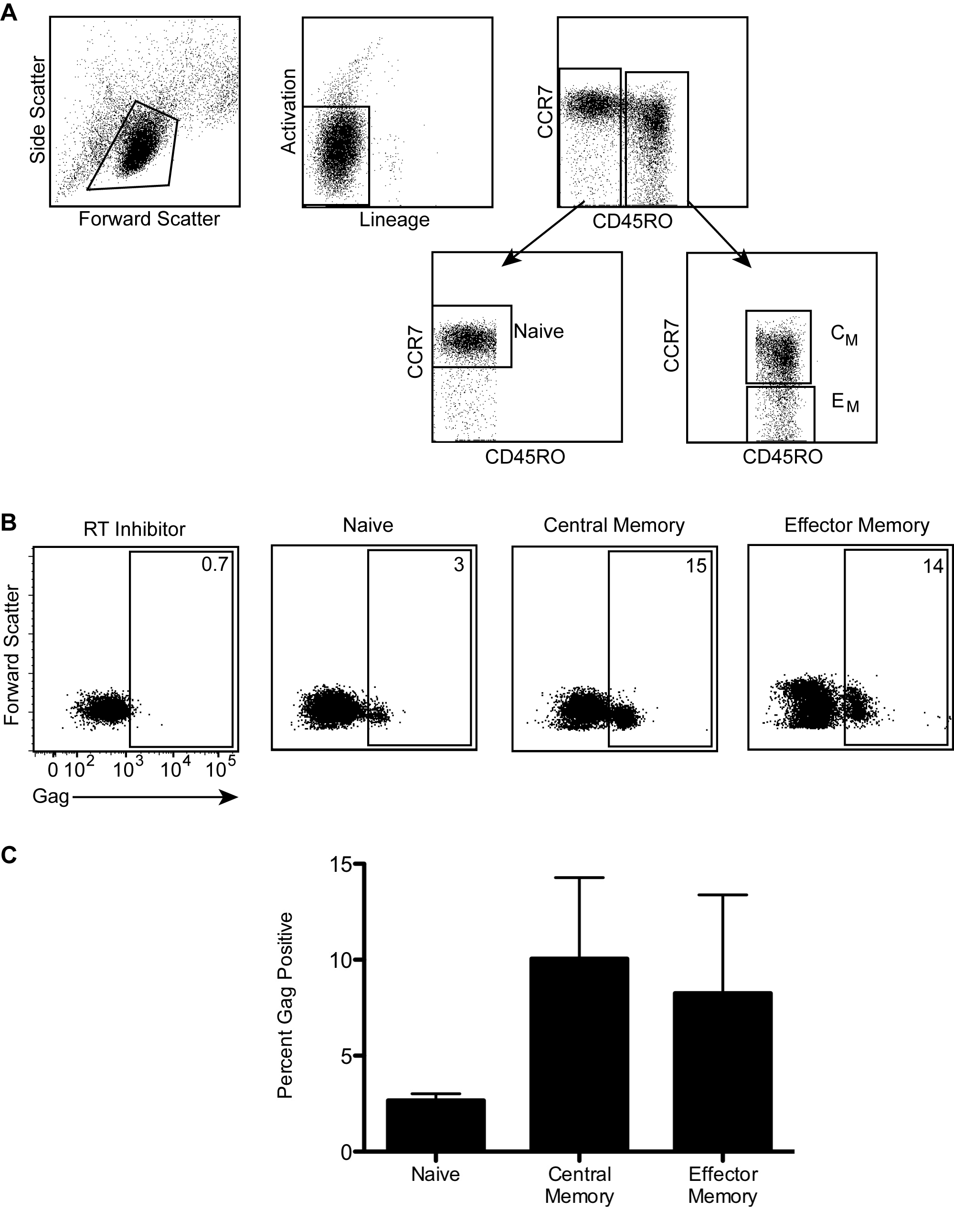

Supplement: Figure S1 — Gag is expressed in several CD4+T cell subsets. Uninfected PBMCs were sorted into resting naïve (CCR7+, CD45RO−), central memory (CM; CCR7+, CD45RO+) and effector memory (EM; CCR7−, CD45RO+) CD4+T cells. Central and effector memory cells were typically >95% pure while naïve cells were typically 99% pure. The CD4+T cell subsets were then spinoculated with NL4-3. Gag expression was measured 72 hours post infection. An efavirenz control was used to determine background levels of Gag. A representative sort strategy is shown in A. A representative experiment is shown in B. In C, an average of 2 experiments in 2 different donors is shown. (TIF) [file ppat.1002818.s001.tif]

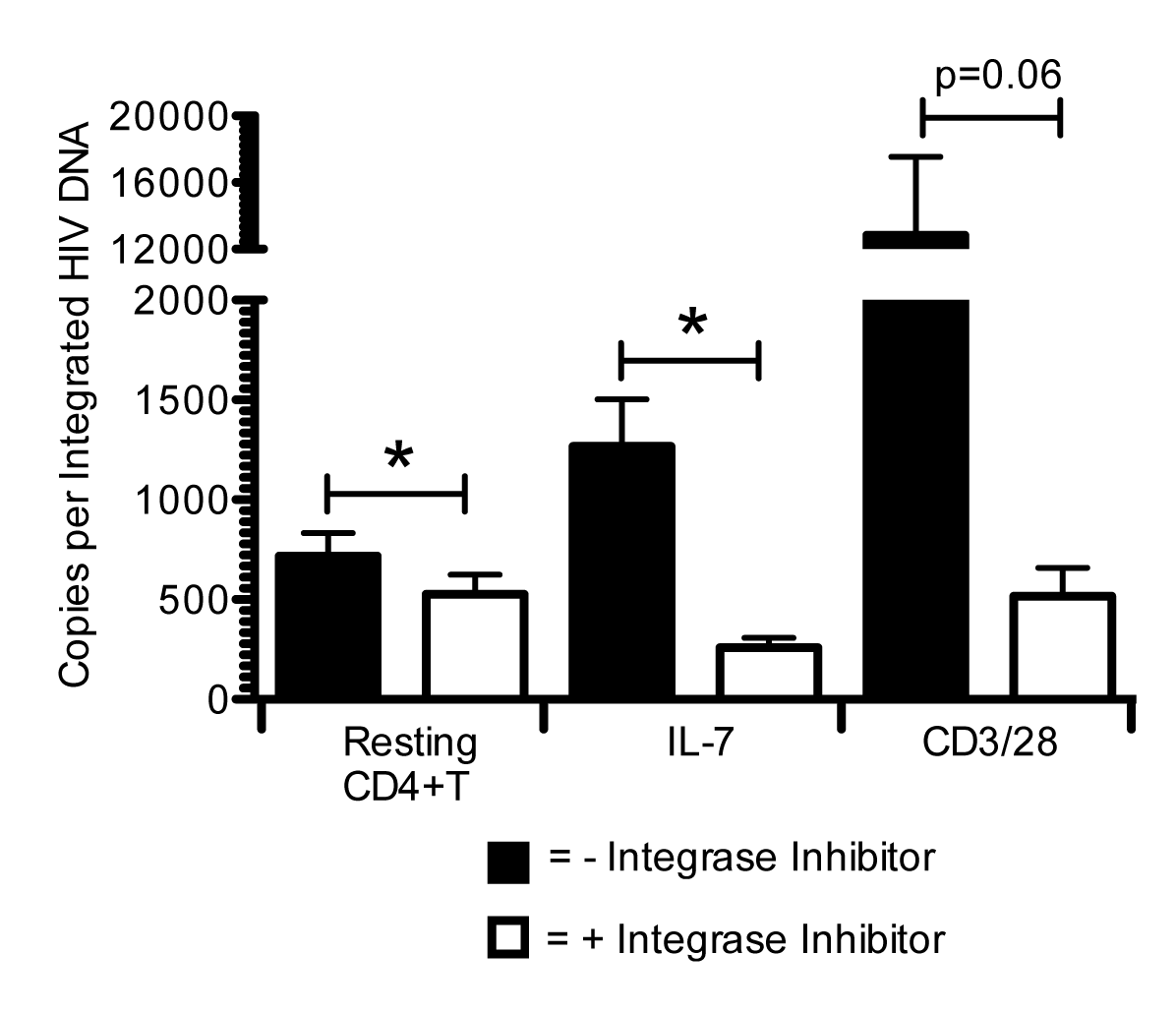

Supplement: Figure S2 — Gag background. Cells were infected and treated as in Figure 4. Levels of RNA in the integrase inhibitor treated and untreated samples are shown. Data are the average of 3 experiments in 3 different donors. The calculated levels of transcribed gag are reported in Figure 5. *Statistically different at the p<0.05 level. (TIF) [file ppat.1002818.s002.tif]
